# Supplementary material for: Multi‐dataset identification of innovative feature genes and molecular mechanisms in keratoconus
Source: J Cell Mol Med. 2024 Sep 19;28(18):e70079. doi: 10.1111/jcmm.70079 (PMC11412914; doi:10.1111/jcmm.70079)
Supplement: Supplementary file 2 — Material S2. [file JCMM-28-e70079-s002.zip › 7.GSEA analysis/4.MYRF/KEGG_SPHINGOLIPID_METABOLISM.html]

Details for gene set KEGG\_SPHINGOLIPID\_METABOLISM[GSEA]

|  || Dataset | MYRF.group.cls#h\_versus\_l |
| Phenotype | group.cls#h\_versus\_l |
| Upregulated in class | l |
| GeneSet | KEGG\_SPHINGOLIPID\_METABOLISM |
| Enrichment Score (ES) | -0.41205922 |
| Normalized Enrichment Score (NES) | -1.4150183 |
| Nominal p-value | 0.06653226 |
| FDR q-value | 0.23204392 |
| FWER p-Value | 0.861 |
Table: GSEA Results Summary

  

Fig 1: Enrichment plot: KEGG\_SPHINGOLIPID\_METABOLISM      
 Profile of the Running ES Score & Positions of GeneSet Members on the Rank Ordered List

  

| SYMBOL | TITLE | RANK IN GENE LIST | RANK METRIC SCORE | RUNNING ES | CORE ENRICHMENT || 1 | UGT8 | na | 2808 | 0.305 | -0.0182 | No |
| 2 | B4GALT6 | na | 5510 | 0.207 | -0.0535 | No |
| 3 | ENPP7 | na | 6438 | 0.180 | -0.0432 | No |
| 4 | ACER1 | na | 7838 | 0.142 | -0.0543 | No |
| 5 | SGPP1 | na | 8477 | 0.127 | -0.0465 | No |
| 6 | ACER3 | na | 8535 | 0.126 | -0.0222 | No |
| 7 | SGPL1 | na | 10898 | 0.080 | -0.0739 | No |
| 8 | ACER2 | na | 11258 | 0.074 | -0.0690 | No |
| 9 | SPHK1 | na | 12659 | 0.054 | -0.0981 | No |
| 10 | NEU4 | na | 14009 | 0.038 | -0.1293 | No |
| 11 | SPTLC2 | na | 14732 | 0.030 | -0.1438 | No |
| 12 | KDSR | na | 15323 | 0.025 | -0.1557 | No |
| 13 | ASAH1 | na | 16706 | 0.011 | -0.1933 | No |
| 14 | NEU3 | na | 16893 | 0.010 | -0.1967 | No |
| 15 | ASAH2 | na | 17044 | 0.008 | -0.1993 | No |
| 16 | SMPD2 | na | 18511 | -0.005 | -0.2405 | No |
| 17 | DEGS2 | na | 18541 | -0.006 | -0.2401 | No |
| 18 | GALC | na | 18848 | -0.009 | -0.2472 | No |
| 19 | ARSA | na | 19989 | -0.022 | -0.2756 | No |
| 20 | NEU1 | na | 20255 | -0.025 | -0.2781 | No |
| 21 | SMPD4 | na | 20460 | -0.027 | -0.2784 | No |
| 22 | SGMS1 | na | 21218 | -0.036 | -0.2929 | No |
| 23 | GLB1 | na | 22760 | -0.056 | -0.3258 | No |
| 24 | GLA | na | 24509 | -0.084 | -0.3589 | No |
| 25 | SPTLC1 | na | 26303 | -0.119 | -0.3861 | Yes |
| 26 | SMPD1 | na | 27118 | -0.138 | -0.3812 | Yes |
| 27 | SPHK2 | na | 28190 | -0.166 | -0.3780 | Yes |
| 28 | CERK | na | 28915 | -0.188 | -0.3601 | Yes |
| 29 | DEGS1 | na | 30574 | -0.244 | -0.3577 | Yes |
| 30 | UGCG | na | 31425 | -0.287 | -0.3232 | Yes |
| 31 | SGMS2 | na | 31781 | -0.306 | -0.2706 | Yes |
| 32 | SMPD3 | na | 32865 | -0.377 | -0.2243 | Yes |
| 33 | NEU2 | na | 33460 | -0.437 | -0.1515 | Yes |
| 34 | SGPP2 | na | 33595 | -0.456 | -0.0617 | Yes |
| 35 | GAL3ST1 | na | 33628 | -0.459 | 0.0317 | Yes |
Table: GSEA details [plain text format]

  

Fig 2: KEGG\_SPHINGOLIPID\_METABOLISM      
 Blue-Pink O' Gram in the Space of the Analyzed GeneSet

  

Fig 3: KEGG\_SPHINGOLIPID\_METABOLISM: Random ES distribution      
 Gene set null distribution of ES for **KEGG\_SPHINGOLIPID\_METABOLISM**
